# Quality of medical prescriptions in diabetes and hypertension management in Kerala and its associated factors

**DOI:** 10.1186/s12889-020-8214-y

**Published:** 2020-02-06

**Authors:** Vijayakumar Krishnapillai, Sanjeev Nair, Anand T. N, Sreelal T. P, Biju Soman

**Affiliations:** 1 Health Action by People, Thiruvananthapuram, Kerala India; 2 Department of Community Medicine, Amrita Institute of Medical Sciences (AIMS), Kochi, Kerala India; 3 Department of Pulmonary Medicine, Government Medical College, Thiruvananthapuram, India; 4 International Decision Support Initiative (iDSI), London, UK; 5 Achutha Menon Centre for Health Science Studies (AMCHSS), SCTIMST, Thiruvananthapuram, India

**Keywords:** Diabetes mellitus, Hypertension, Kerala, Non-communicable diseases, Prescription, Quality

## Abstract

**Background:**

Kerala is facing challenges in the secondary prevention efforts of non-communicable diseases (NCDs). In spite of being the top performer in health parameters among Indian states, the burden of NCDs, especially diabetes mellitus (diabetes) and hypertension, is higher in Kerala. This research endeavours to identify the role of quality of medical prescriptions in secondary prevention of diabetes and hypertension and suggest corrective measures.

**Methods:**

This cross-sectional study involved collection of prescription data and other details from consenting doctors across seven districts in Kerala. After the quality of prescription was assessed using a checklist, scores were generated, and cutoff points were used to classify the prescriptions. PASW version 18 software, was used for data analysis which included univariate and bivariate analyses and logistic regression. The proportion of quality prescriptions was estimated after adjusting for clustering, and the proportion of doctors writing quality prescriptions was also estimated. Prior to the study, ethical clearance from Independent ethics committee in Health action by People (HAP) and informed consent from all the study participants were obtained.

**Results:**

After assessing 9199 prescriptions from 344 doctors, it was found that about 37.2% (95% CI: 34.9–39.4%) of the prescriptions were of good quality, and 48.2% (95% CI: 42.9–53.7%) of the doctors provided quality prescriptions. Factors associated with quality prescriptions were found to be knowledge about NCD guidelines, quality certifications of hospitals and usage of patient data management software.

**Conclusions:**

In the context of rising prevalence of NCDs and the challenges in the secondary prevention efforts, this is one of the first studies in Kerala to evaluate the quality of prescriptions to manage NCDs as prescriptions often reflect the quality of medical management. The study also addresses other factors associated with quality medical management. The findings indicate that the scope for improvement is more than 50%, when considered for the overall quality of prescriptions in diabetes and hypertension management. Further, it was found that appropriate training of doctors, adherence to treatment guidelines and the use of technology may improve the overall quality of prescriptions.

## Background

Kerala’s health indicators are comparable to those of developed countries and [1] it is the top performing state in India in terms of health. This achievement was made possible due to its unique health system [2–4]. The health system in Kerala includes both government/public hospitals and private hospitals. The public health system works at three levels: sub-centres and primary health centers at the primary level, community health centres and sub-district hospitals at the secondary level, and district, regional and medical college hospitals at the tertiary level. In the private sector, general practitioners and small clinics operate at the primary level, and medium and larger specialty hospitals function at the secondary and the tertiary levels. In spite of all the achievements of Kerala in the health sector, the burden of non-communicable diseases (NCDs), especially diabetes (diabetes mellitus) and hypertension, is higher here compared to other states in India. At present, medical professionals and the health care system are in an ideal position to help patients with NCDs. They are playing a crucial role in linking pharmacological and non-pharmacological methods for secondary prevention of NCDs.

Medical prescriptions play an important part in the management of non-communicable diseases. Medical prescription is a non-verbal channel which communicates medication plans from prescribers to pharmacists and patients. A good prescription should be rational, evidence based, clear, complete, and should be able to improve the treatment outcome of a patient [5]. Improper prescribing patterns include a prescription without an appropriate indication, correct dose, frequency, route of administration, schedule or duration of treatment; prescribing duplicate therapeutic agents, and medication with potential drug–drug interactions or adverse reactions are also considered to be improper pattern of prescription [6]. Prescriptions of poor quality can lead to inappropriate uses of medicines and ineffective treatment outcomes [7]. The prescription patterns of physicians are powerful tools to ascertain the role of drugs in society.

However, there is uncertainty among physicians regarding the selection of drugs because various trials are coming out with divergent results, and various guidelines reviewing the contemporary literature are arriving at different conclusions. Therefore, the secondary prevention efforts of diabetes and hypertension in the state are facing challenges which have resulted in high cardiovascular morbidity and mortality.

According to PROLIFE study conducted in Kerala, the age-standardized cardiovascular disease death rates were 490 for men and 231 for women per 100,000 person years [8]. This finding communicates the need for reassessing the secondary prevention strategies of the state. Hence, there is a need for studying the pattern of drug prescriptions for diabetes and hypertension management, along with the assessment of subjective and objective factors that affect doctors’ prescription patterns. Quality assessment of prescriptions and adherence to NCD management guidelines will help to increase prescription quality, improve the rationality of drug use, to identify the problems in secondary prevention of NCD, and take corrective actions. Therefore, our objective was to assess the quality of prescriptions offered by doctors in diabetes and hypertension management in Kerala and to find out the proportion of doctors offering quality prescriptions along with the associated factors.

## Methods

This is a cross-sectional study conducted in 7 districts (Thiruvananthapuram, Kottayam, Waynad, Alappuzha, Ernakulam, Kozhikode, Kasargod) that were randomly selected from 14 districts of Kerala. Further, a sample size of 333 doctors was calculated (with 95% confidence level, relative precision of 20% and a design effect of 1.5) on the assumption that 30% of doctors/physicians gave “quality prescriptions” (based on the results of a student research project conducted in the Department of Community Medicine, Medical College, Trivandrum).

Primary and secondary care level health institutions were included in the study. 182 public sector institutions from 7 districts were randomly selected from a list of 499 institutions obtained from Directorate of Health Services (DHS), Kerala. Also, 162 private clinics and hospitals were selected randomly from a list of institutions prepared from these districts. Only doctors who are specialists in managing NCDs or who are managing NCD cases more frequently were considered for the study. One consenting doctor from each institution was included in the study. Further, ethical clearance was obtained from Independent ethics committee in Health Action by People (HAP). Written informed consent was obtained from all the study participants, and prescription data collected were masked from personal identification information.

Information on doctors and institutional characteristics was collected using a questionnaire. A *new tool or checklist* (Additional file 1) was developed in consultation with an expert committee and by reviewing other available prescription quality tools. It contained 19 items under different quality domains - General prescription quality (3 items), Patient information quality (5 items), Pharmacological intervention quality (6 items – which included 4 items for diabetes only, 2 items for hypertension only and all 6 items for hypertension with diabetes), Lifestyle modification advice (5 items). Item scores or ratings were decided based on the priority mentioned by the expert panel.

After these preparations a pilot study was conducted in selected private clinics and primary health centers (PHCs) in Thiruvananthapuram district and based on its observations, the data collection process was revised. Soon, trained field data collectors contacted the consenting doctors in the selected institutions and collected information using a questionnaire. The field data collectors also took photographs of prescriptions (diabetes/hypertension/ diabetes with hypertension) given by the doctors using tablets, and digital copies of prescriptions were collected. However, identification details of patients and institutions were not collected. At least 25 prescriptions were collected from each doctor and communication was maintained with each doctor to ensure data quality. Both retrospective and prospective prescriptions were collected. All the prescriptions were ± 6 months from the date on which consent was obtained. A Research Fellow assessed the data for any errors and a qualified pharmacist assisted in data entry from the prescriptions.

In the next stage, all prescriptions were assessed using the checklist (Additional file 1) and aggregated scores were generated for each prescription. Good/quality prescriptions were selected using the predetermined cutoff points or scores developed in consultation with experts and in comparison with previous studies. The minimum scores required for a quality prescription was 11 out of 27 (maximum score) for diabetes mellitus, 9 out of 22 in hypertension and 15 out of 33 for hypertension with diabetes mellitus cases. From each consenting doctor in the study 25 prescriptions were assessed and a doctor was considered to be providing quality prescriptions, if more than 40% prescriptions were of good quality.

Statistical analysis: Data entry and analysis were done using Epi-data version 3.1and PASW version 18 respectively. The unit of analysis was doctors. Mean and standard deviation were used to summarize continuous variables frequencies and proportions were used to summarize categorical variables. While calculating the proportion of quality prescriptions, the clustering effect was taken into account and adjusted for clustering. Bivariate analysis was done to estimate Odds ratios and 95% confidence intervals. Logistic regression analysis was done to estimate adjusted odds ratios and confidence intervals; here dependent variable was doctors issuing quality prescriptions. The variables which were statistically significant in bivariate analysis were selected as independent variables.

## Results

Prescription practice of 344 doctors was assessed based on 9199 prescriptions collected from them. Proportion of doctors issuing good quality prescriptions as well as proportion of good quality prescriptions in the study was assessed. The study found that 48.2% (95% confidence interval (95% CI) 42.9–53.7%), *n* = 166, of the doctors were writing good/quality prescriptions: 58.3% (95% CI: 51.0–65.6%), *n* = 102, in the public sector and 37.9% (95% CI: 30.6–45.2%), *n* = 64 in the private sector. A rural-urban difference was also observed: 52.3% (95% CI: 46.3–58.3%), *n* = 139, of doctors in rural areas gave quality prescription compared to only 34.6% (95% CI: 24.1–45.2%), *n* = 27, in urban areas.

Looking at the details, a total of 9199 prescriptions of NCDs were collected which included prescription of hypertension *n* = 3373 (36.7%), diabetes *n* = 2730 (29.7%), and diabetes with hypertension *n* = 3096 (33.7%). The proportion (Crude) of good/quality prescriptions was 37.2% (*n* = 3419) (95% CI of 36.2–38.2%); however, after adjusting for clustering, the proportion of quality prescriptions was 37.2% (95% CI: 34.9–39.4%) which was arrived at after standard error was calculated using adjusted analysis for clusters. When the proportion of quality prescriptions for the selected diseases was calculated it was 79.6% (95% CI: 78.2–80.9%) (*n* = 2684) in hypertension, 16.8% (95% CI: 15.4–18.3%) (*n* = 460) in diabetes and 8.9% (95% CI: 7.9–9.9%) (*n* = 275) in diabetes with hypertension. Other relevant findings were that diagnosis was written only in 25% prescriptions, a majority of the prescriptions contained details such as date, dose, route and timings, patient age, medications and only few prescriptions had life style modification advice namely, cessation of tobacco and alcohol consumption, weight reduction, salt restriction and increasing physical activity (Table 1).

**Table 1:** The domains, items and findings from the prescription quality checklist. (*N* = 9199)

Domains	Items	Availability N (%)
General prescription quality	1. Date of prescription	8444 (91.8)
	2. Dose, route and timings clearly mentioned	9154 (99.5)
	3. Medical Diagnosis mentioned	2298 (25.0)
Patient information quality	4. Patient Age mentioned	8709 (94.7)
	5. Patient Gender	5157 (56.1)
	6. Patient Height/Weight/BMI	365 (4.0)
	7. Patient Blood Pressure	5268 (57.3)
	8. Patient Blood sugar/HbA1c	3178 (34.5)
Medication/Pharmacological intervention quality	Diabetes (*N* = 2730)	
	9. “Metformin” prescribed	2103 (77.0)
	10. Statins prescribed	458 (16.8)
	11. ACEI/ARB prescribed	9 (0.3)
	12. Use of more than one drug from same group	48 (1.8)
	Hypertension (*N* = 3373)	
	13. Is patient on 24 h antihypertensive	3196 (94.8)
	14. ACEI/Beta Blocker/CCB/Thiazide Diuretics	3196 (94.8)
	Diabetes with Hypertension 9–14 (*N* = 3096)	
	“Metformin” prescribed	2257 (72.9)
	Statins prescribed	889 (28.7)
	ACEI/ARB prescribed	1387 (44.8)
	Use of more than one drug from same group	9 (3.1)
	Is patient on 24 h antihypertensive	2887 (93.2)
	ACEI/Beta Blocker/CCB/Thiazide Diuretics	2887 (93.2)
Lifestyle modification advice/Additional Points	15. Tobacco cessation advice	5 (0.1)
	16. Weight reduction	17 (0.2)
	17. Alcohol consumption cessation	6 (0.1)
	18. Salt restriction	5 (0.1)
	19. Improving physical activity	5 (0.1)

Considering prescriptions which had results of basic investigations such as blood pressure and results of blood tests, it was calculated that 57.3% (*n* = 5268) of all the prescriptions had such information. To be specific, such information was present in 70.5% (*n* = 2377) of hypertension prescriptions, 59.4% (*n* = 1839) of diabetes with hypertension prescriptions and 38% (*n* = 1052) of the diabetes prescriptions. Results of blood glucose tests were mentioned in 34.5% (*n* = 3178) prescriptions, which included FBS (*n* = 1387), PPBS (*n* = 526), RBS (*n* = 1175), HbA1C (*n* = 6). Further, blood glucose test results were mentioned in 46.2% (*n* = 1262) diabetes prescriptions and 44.3% (*n* = 1370) of diabetes with hypertension prescriptions, but it was low in hypertension prescriptions 16.2% (*n* = 546). Anthropometric measurements were mentioned only in a few prescriptions [4% (*n* = 365)] (Table 1), which included 5.3% (*n* = 179) in hypertension, 4.2% (*n* = 129) in Diabetes with hypertension and 2.1% (*n* = 57) in diabetes.

Regarding the use of statins, it was found that 21.8% (*n* = 2009) prescriptions had statins, and Atorvastatin was the most commonly prescribed statin, 91.2% (*n* = 1834). However, there was no major difference in the prescription of statins with the quality of prescriptions 21.3% (*n* = 727) in good quality prescriptions versus 22.2% (*n* = 1282) in poor quality prescriptions) (*p* < 0.001). It is also noticeable that statins were prescribed almost equally in public [21.7% (*n* = 1089)] and private [22.0% (*n* = 920)] institutions, (p < 0.001). One remarkable finding is that statins were prescribed more in case of diabetes with hypertension 28.7% (*n* = 889) compared to diabetes, 16.8% (*n* = 458) or hypertension, 19.6% (*n* = 662) alone, and this was statistically significant (*p* < 0.001).

Considering the most commonly prescribed drugs, in diabetes mellitus (diabetes only or with hypertension), it was Metformin 67.8% (*n* = 3951), followed by Glimepiride 33.4% (*n* = 1946) and insulin which was prescribed to 19.8% (*n* = 1153) patients (Table 2). For patients with hypertension (hypertension alone or with diabetes mellitus), Amlodipine 50.7% (*n* = 3282) was the most preferred antihypertensive drug, followed by Atenolol 22.0% (*n* = 1426) (Table 3).

**Table 2:** Drugs prescribed for diabetic patients (Diabetes and Diabetes with hypertension)

Variables	Diabetes (*N* = 2730)		Diabetes with Hypertension (*N* = 3096)		Total (*N* = 5826)	
Antidiabetic drugs	n	%	n	%	N	%
Metformin	1893	69.3	2058	66.5	3951	67.8
Glimepride	987	36.2	959	31.0	1946	33.4
Glibenclamide	557	20.4	795	25.7	1352	23.2
Inj Human Mixtard	576	21.1	577	18.6	1153	19.8
Acarbose	210	7.7	143	4.6	353	6.1
Pioglitazone	138	5.1	54	1.7	192	3.3
Gliclazide	63	2.3	104	3.4	167	2.9

**Table 3:** Drugs prescribed for hypertensive patients (hypertension and diabetes with hypertension)

Variables	Hypertension (*N* = 3373)		Diabetes with Hypertension (*N* = 3096)		Total (*N* = 6469)	
Antihypertensives	n	%	n	%	N	%
Amlodipine	1842	54.6	1440	46.5	3282	50.7
Atenolol	753	22.3	673	21.7	1426	22.0
Losartan	711	21.1	649	21.0	1360	21.0
Telmisartan	293	8.7	477	15.4	770	11.9
Hydrochlorothiazide	187	5.5	166	5.4	353	5.5
Enalapril	115	3.4	154	5.0	269	4.2
Ramipril	18	0.5	43	1.4	61	0.9
Lasix	16	0.5	26	0.8	42	0.6

As regards the association between quality of prescriptions and disease control status, it was found that in patients with diabetes only, glycemic control was high in the presence of good quality prescription 47.0% compared to poor quality prescription 34.8% and this was significant (*p*-value = 0.002) (Tables 4 & 5). On the contrary, in patients with hypertension only, the poor quality prescription group showed a higher proportion of BP control (38.5%) compared to good quality prescription 30.2%. However, in diabetes with hypertension group, there was no significant association between disease control status and quality of prescriptions.

**Table 4:** Comparison of Blood Pressure control status with the Quality of Prescriptions among patients with hypertension or Diabetes with hypertension

Disease category	BP control status	Quality of Prescriptions		Total	*p*-value*
		Good n(%)	Poor n(%)		
Diabetes with hypertension	Controlled	83 (39.9)	542 (33.3)	625 (34.1)	0.059
	Not Controlled	125 (60.1)	1085 (66.7)	1210 (65.9)	
	Total	208 (100)	1627 (100)	1835 (100)	
Hypertension	Controlled	605 (30.2)	144 (38.5)	749 (31.5)	0.002
	Not Controlled	1396 (69.8)	230 (61.5)	1626 (68.5)	
	Total	2001 (100)	374 (100)	2375 (100)	

^*^Chi-square.

**Table 5:** Comparison of blood glucose control status with the Quality of Prescriptions among patients with diabetes or diabetes with hypertension

Disease category	Blood glucose control status	Quality of Prescriptions		Total n(%)	*p*-value*
		Good n(%)	Poor n(%)		
Diabetes with hypertension	Controlled	22 (61.1)	604 (46.6)	626 (47.0)	0.085
	Not Controlled	14 (38.9)	692 (53.4)	706 (53.0)	
	Total	36 (100)	1296 (100)	1332 (100)	
Diabetes	Controlled	85 (47.0)	363 (34.8)	448 (36.6)	0.002
	Not Controlled	96 (53.0)	681 (65.2)	777 (63.4)	
	Total	181 (100)	1044 (100)	1225 (100)	

*Chi-square

In bivariate analysis, the significant factors that were associated with writing of good quality prescriptions by doctors were found to be age of the doctors, number of years of experience, number of patient consultation per day, hospital type, hospital location, awareness about NCD guidelines, quality certification and availability of software for patient data management (Table 6). However, after logistic regression analysis, only quality certification of the hospitals, awareness of NCD guidelines and usage of software for patient data management was found to be the significant factors (Table 6).

**Table 6:** The factors associated with doctors issuing good/quality prescriptions (*n* = 344)

Variable	Category	Total number of doctors	Doctors issuing quality prescriptions (*n* = 166)	OR (95% CI)	Adjusted OR (95% CI)	*p*-value
		*N* = 344	n (%)			
Age group (years)	20–40 (yrs)	176	95 (54.0)	1.6 (1.1–2.5)	1.2 (0.7–2.1)	0.42
	41–80 (yrs)	168	71 (42.3)			
Experience (years)	0–30 yrs	290	149 (51.4)	2.3 (1.2–4.3)	1.1 (0.5–2.3)	0.82
	31–60 yrs	54	17 (31.5)			
Patient Consultation (number/day)	0–80 /day	176	68 (38.6)	0.4 (0.3–0.7)	0.6 (0.4–1.1)	0.08
	81–250/day	168	98 (58.3)			
Hospital type	Public	175	102 (58.3)	2.3 (1.5–3.5)	1.6 (0.9–3.0)	0.11
	Private	169	64 (37.9)			
Hospital Location	Rural	266	139 (52.3)	2.1 (1.2–3.5)	1.8 (0.9–3.4)	0.056
Awareness on NCD guide	Yes	218	118 (54.1)	1.9 (1.2–3.0)	1.8 (1.04–2.9)	0.03
	No	126	48 (38.1)			
Quality certification	Yes	51	33 (64.7)	2.2 (1.2–4.1)	2.2 (1.1–4.4)	0.02
	No	293	133 (45.4)			
Software for patient data management	Yes	26	22(84.6)	6.6 (2.2–19.7)	12.1 (3.8–38.5)	< 0.001
	No	318	144 (45.3)			
Gender	Male	203	91 (44.8)	0.7 (0.5–1.1)	–	–
	Female	141	75 (53.2)			
Marital status	Married	313	149 (47.6)	0.7 (0.4–1.6)	–	–
	Single	31	17 (54.8)			
Education	PG	167	73 (43.7)	0.7 (0.5–1.1)	–	–
	MBBS	177	93 (52.5)			
Place of education	Domestic	336	161 (47.9)	0.6 (0.1–2.3)	–	–
	International	8	5 (62.5)			
Number of CMEs attended	One CME	236	118 (50)	1.3 (0.8–1.9)	–	–
	No CME	108	48 (44.4)			
Journal Subscription	Yes	96	47 (49.0)	1.0 (0.6–1.7)	–	–
	No	248	119 (48.0)			
Consultation time (hrs)/day	0–6 h	168	76 (45.2)	0.8 (0.5–1.2)	–	–
	7–11 h	176	90 (51.1)			
Contract Type	Permanent	269	136 (50.6)	1.5 (0.9–2.6)	–	–
	Temporary	75	30 (40.0)			
Clinical research	Yes	39	20 (51.3)	1.1 (0.6–2.2)	–	–
	No	305	146 (47.9)			
	Urban	78	27 (34.6)			
Pharmacy availability	Yes	327	159 (48.6)	1.4 (0.5–3.6)	–	–
	No	17	7 (41.2)			

Variable(s) entered in logistic regression are – age group, experience of doctor, Patient consultation per day, hospital type, hospital location, awareness of NCD guidelines, quality certification, software for patient management.

## Discussion

There are two main findings of this study. Firstly, over one-third of the prescriptions for NCD management were of good quality and nearly half of the doctors gave quality prescriptions to their patients with NCDs which included diabetes, hypertension or both. Secondly, factors such as doctors` training status, adherence to NCD guidelines, institutional quality assurance and the role of prescribing software in reducing errors in prescriptions have had a major impact on prescription quality. This has been stated elsewhere as an important component of evidence informed practice [9].

On analyzing the quality of prescriptions, the majority of the prescriptions for hypertension were found to be of good quality. This is in contrast to the study done by Suthar J and Patel V, who had used prescription quality index as a tool to measure quality of prescriptions [10]. However, in the case of diabetes with hypertension, the proportion of quality prescriptions was low. This may be attributed to the complexity of treatment, including the stages of the disease, any comorbidities and number of medications required. Only one-fourth of the prescriptions had an accurate diagnosis unlike what a few other studies had found [11, 12].

Metformin for diabetes mellitus and amlodipine for hypertension were the most common drugs prescribed. In the anti-diabetic prescriptions, most common oral hypoglycemic agent (OHA) prescribed was metformin (biguanide group) followed by glimipride (sulphonyl urea) as reported in other studies from South India [13–15]. A few studies in tertiary care settings in India also showed similar findings, but the proportions were low [14, 15]. The standard guidelines recommend the use of metformin as the first choice antidiabetic for patients with type 2 diabetes mellitus [16]. Similar to other studies [15], insulin accounts for about one-fifth of the anti-diabetic prescriptions. For blood pressure (BP) management, the JNC guidelines recommend Thiazide diuretics as a first-line drug, either alone or in conjunction with other groups of antihypertensive drugs [17]. In this study, Thiazide diuretic prescription was only minimal whereas amlodipine (calcium channel blocker) was the most prescribed anti-hypertensive medication followed by atenolol (beta blocker). This may be due to the presence of patients who were at various stages of the disease.

Further, though in this study only one-fifth of the prescriptions for hypertension contained statins, there are other multi-site prescription studies in India that have recorded more than 50% statin prescription [18]. Even though many studies have shown the effect of statins on lowering of blood pressure, the present study shows that statin prescription in Kerala is lower than that found in other studies [19, 20].

It was found that very few prescriptions contained advice on lifestyle changes, such as advice on cessation of tobacco use and alcohol consumption, restriction of salt, increased physical activity, and weight reduction. This may be due to less attention provided to the behavior change component in therapy or may be because such advice are offered as recommendations (verbally) rather than written prescriptions. Hypertension and diabetes are closely linked to a person’s lifestyle, and lifestyle modification is an important part of NCD management [21–23]. In diabetes with hypertension group, there was no significant association between the disease control and the quality of prescriptions. In only diabetes patients’ glycemic control was significantly better in the presence of good quality prescriptions compared to the poor quality prescriptions.

Although there are many studies evaluating the presence of lifestyle advice during consultation, no studies could be found on the proportion of doctors who issued quality prescriptions in NCD management. In this study, the factors which were significantly associated with good prescription behavior in doctors were availability of the NCD treatment guideline in the hospital, quality certification of the hospital and use software for prescription.

Knowledge of NCD guidelines has been found to increase the likelihood of doctors prescribing good quality prescriptions. A similar study conducted in tuberculosis management also showed the same findings [24]. The study findings show that doctors in the public sector provided better quality prescriptions than doctors in the private sector, which may be due to a lack of adherence to standard guidelines in the private system. NCD guidelines issued by the state government are available in the public hospitals, but these guidelines are not available in many of the private clinics; this emphasizes the need to incorporate the private health care system in the NCD management program of the state.

Likewise, doctors tend to provide more quality prescriptions for NCDs in institutions with a quality assessment system. Previous studies also indicate that enhancing the competence of doctors, strengthening adherence to guidelines and performance evaluation [25] would improve quality prescribing behavior. In this study, usage of software for prescription was also associated with good prescription pattern.

Many studies have also shown that use of electronic prescribing systems in hospitals can help to reduce prescription errors. This may improve the quality of prescriptions as well as quality of care [26, 27], but this requires sufficient funding and extensive training of doctors on the usage of electronic system.

Factors such as age of doctors, years of experience, number of patients consulted per day, location of hospitals, and type of hospitals showed no significant association after adjusting for the confounders. Some studies have shown a positive association between uptake of a newer drug and doctor’s age, but there is no relevant study stating the association with quality of prescription in NCD management [25]. A systematic review has shown that doctors with more clinical experience may be at risk of non-compliance with standards of care for therapy and thus may provide poor quality care [28]. Similar results have been found in this study, with doctors with fewer years of experience giving more quality prescription. The reason may be that they are updated on latest guidelines and are willing to adopt newer drugs. Doctors who consult more patients are associated with quality prescription behavior; this indicates that patients meet doctors who give quality care. Doctors who participate in continuing medical education, frequently read scientific journals and have experience in clinical research provided quality prescriptions compared to their counterparts who did not engage in these activities. However, these results were not found to be statistically significant in this study, which could be due to a lack of adequate sample size.

There were some limitations of the study. A significant one was that the prescription quality was assessed using a checklist developed solely based on expert consultation, and the patient side data were not collected. The findings were therefore less representative of the factors associated with the patients. Another major limitation was that there is a potential chance of bias from some participating doctors altering their prescribing behavior, consequent to an awareness of being part of the study, but this bias may be minimal since we have some retrospective as well as prospective prescription data. Moreover, some lifestyle modification advice might have been given verbally, and that part is lacking in the prescription analysis. Finally, the sample size was estimated to capture the proportion of doctors issuing good quality prescriptions, so the sample may not be adequate to identify the factors associated.

## Conclusion

With heavy burden of non-communicable diseases and its complications, Kerala is facing serious problems in the secondary prevention efforts of diabetes and hypertension. As medical prescriptions are an important part of medical management of NCDs,periodic quality assessment, corrections based on the findings of the assessment and adherence to medical management guidelines will help to improve the rationality of drug use, to identify issues related to secondary prevention of NCDs, and to take corrective actions.

This is one of the first studies to evaluate the quality of prescriptions in diabetes mellitus and hypertension management in Kerala. Prescriptions from both public and private care providers were subjected to quality assessment. The findings show the need to improve the quality of prescriptions issued by doctors. The policy implications are the following: There is a need to periodically publish guidelines for diabetes mellitus and hypertension management and provide to doctors in both public and private sectors. The training for doctors should be extended to both public and private sectors. The modern technology such as electronic prescribing systems should be used to reduce prescription errors and to improve quality of care. Similarly, continuous monitoring of the quality and adequate corrections on guidelines are essential for improving the medical management.

## Supplementary information

**Additional file 1.** PRESCRIPTION QUALITY ASSESSMENT CHECKLIST.

## Data Availability

The data sets used and/or analyzed during the current study are available from the corresponding author on reasonable request.

## Abbreviations

BP: Blood pressure
CME: Continuing Medical Education
DHS: Directorate of Health Services
FBS: Fasting Blood Sugar
HAP: Health action by People
ICMR: Indian Council of Medical Research
JNC: Joint National Committee on Hypertension
LSM: Lifestyle modification
NCD: Non-communicable disease
OHA: Oral hypoglycemic agent
PASW: Predictive Analytics SoftWare
PHC: Primary health centers
PPBS: Post Prandial Blood Sugar
PROLIFE: Population Registry of Lifestyle Diseases
RBS: Routine Blood Sugar
