# Supplementary material for: Quality of medical prescriptions in diabetes and hypertension management in Kerala and its associated factors
Source: BMC Public Health. 2020 Feb 6;20:193. doi: 10.1186/s12889-020-8214-y (PMC7006375; doi:10.1186/s12889-020-8214-y)
Supplement: Supplementary file 1 — Additional file 1. PRESCRIPTION QUALITY ASSESSMENT CHECKLIST. [file 12889_2020_8214_MOESM1_ESM.doc]

# PRESCRIPTION QUALITY ASSESSMENT CHECKLIST

| Doctor id |  |
| --- | --- |
| **Institution type** | **Public** |
|  | **Private** |
| **District id** |  |
| **DATE:** |  |
| **REVIEWED BY** |  |

| Disease condition | | **Diabetes** | |  | | | |
| --- | --- | --- | --- | --- | --- | --- | --- |
|  |  | **Hypertension** | |  | | | |
|  |  | **Diabetes & Hypertension** | |  | | | |
|  |  | | | | | | |
| RATING | | | Score | | | Y | **N** |
|  |  |  | Y | | N |  |  |
| General quality | | |  | |  |  |  |
| Date of prescription | | | **1** | | **0** |  |  |
| Dose route and timings clearly mentioned | | | **2** | | **0** |  |  |
| Medical Diagnosis mentioned | | | **2** | | **0** |  |  |
| Patient details | | |  | |  |  |  |
| 1. Patient Age mentioned | | | 1 | | 0 |  |  |
| 1. Patient Gender | | | 1 | | 0 |  |  |
| 1. Patient Height/Weight/BMI | | | 2 | | 0 |  |  |
| Patient Blood Pressure | | | 2 | | 0 |  |  |
| Patient Blood sugar/ HbA1c | | | 2 | | 0 |  |  |
| MEDICATION | | |  | |  |  |  |
| **DIABETES** | | |  | |  |  |  |
| 1. “Metformin” prescribed | | | 5 | | 0 |  |  |
| 1. Statins prescribed | | | 2 | | 0 |  |  |
| 1. ACEI/ARB prescribed | | | 2 | | 0 |  |  |
| 1. Use of more than one drug from same group | | | -1 | | 0 |  |  |
| **HYPERTENSION** | | |  | |  |  |  |
| 1. Is patient on 24 hr antihypertensive | | | 5 | | 0 |  |  |
| 1. ACEI/BETA BLOCKER/ CCB /THIAZIDE DIURETICS | | | 1 | | 0 |  |  |
| **DIABETES with HYPERTENSION 9-14** | | |  | |  |  |  |
| **ADDITIONAL POINTS** | | |  | |  |  |  |
| 1. Tobacco cessation advice | | | 1 | | 0 |  |  |
| 1. Weight reduction | | | 1 | | 0 |  |  |
| 1. Alcohol consumption cessation | | | 1 | | 0 |  |  |
| 1. Salt restriction | | | 1 | | 0 |  |  |
| 1. Improving physical activity | | | 1 | | 0 |  |  |

| **No** | **Disease** | **Maximum score** | **Minimum score** | **Minimum score for quality prescription** |
| --- | --- | --- | --- | --- |
| **1.** | **Diabetes** | **27** | **-1** | **11 (with 9 Yes)** |
| **2.** | **Hypertension** | **22** | **0** | **9 (with 13 Yes)** |
| **3.** | **Diabetes and hypertension** | **33** | **-1** | **15 (with9&13 Yes)** |
